# Thermodynamic and Kinetic Simulations Used for the Study of the Influence of Precipitates on Thermophysical Properties in NiTiCu Alloys Obtained by Spark Plasma Sintering

**DOI:** 10.3390/nano14050461

**Published:** 2024-03-02

**Authors:** Cristiana Diana Cirstea, Erwin Povoden-Karadeniz, Vasile Cirstea, Felicia Tolea, Ernst Kozeschnik

**Affiliations:** 1 National Institute for Research and Development in Electrical Engineering ICPE-CA (INCDIE ICPE-CA), Splaiul Unirii 313, 030138 Bucharest, Romania; 2 Christian Doppler Laboratory for Interfaces and Precipitation Engineering CDL-IPE, Institute of Materials Science and Technology, TU Wien, Getreidemarkt 9, 1060 Vienna, Austria; erwin.povoden-karadeniz@tuwien.ac.at; 3 Institute of Materials Science and Technology, TU Wien, Getreidemarkt 9, 1060 Vienna, Austria; ernst.kozeschnik@tuwien.ac.at; 4 Military Equipments and Technology Research Agency, Aeroportului 16, 077025 Clinceni, Romania; cvasile76@gmail.com; 5 National Institute of Materials Physics (INCDFM), 105 bis Atomistilor Str., 077125 Magurele, Romania; felicia.tolea@infim.ro

**Keywords:** thermodynamic computation, shape memory, martensitic transformation, thermal hysteresis, thermal conductivity

## Abstract

The thermodynamic and kinetic simulations based on the re-assessment of the thermodynamic and kinetic database of the Ni-Ti-Cu system were employed to predict the phenomena of mechanical alloying, spark plasma sintering and thermal properties of the intriguing Ni-Ti-Cu system. Thermodynamic calculations are presented for the stable and unstable phases of NiTiCu materials and support a correlation with the evolving microstructure during the technological process. Also, the thermal conductivity, the thermal diffusivity and the specific heat of spark plasma sintered and aged Cu-alloyed NiTi-based shape memory alloys (NiTiCu) with two compositions, Ni_45_Ti_50_Cu_5_ and Ni_40_Ti_50_Cu_10_, are evaluated and the influence of mechanical alloying and precipitates on thermal properties is discussed. Measurements of these thermal properties were carried out from 25 °C up to 175 °C using the laser flash method, as well as differential scanning calorimetry. The thermal hysteresis of the 20 mm diameter samples was between 8.8 and 24.5 °C. The observed *T*_0_ temperatures from DSC experimental transformation features are in reasonable accordance with the thermodynamic predictions. The determined *k* values are between 20.04 and 26.87 W/m K and in agreement with the literature results. Moreover, this paper can provide some suggestions for the preparation of NiTiCu shape memory alloys and their applications.

## 1. Introduction

Over the recent years, many industries depended on material advancements to bring improved technologies to market. Thus, using a computational approach can help us to design new materials that are complementary to traditional experimental-based materials discovery. One major advantage of computations is the level of control they offer compared to experiments. For example, during the improvement of a material or the designing of a new one, it is often necessary to simulate the effects of chemical substitutions, microstructural phases or thermal and mechanical properties of materials, but achieving those same conditions experimentally could take many months of laboratory work. In addition, characterizing a material’s fundamental properties is more quickly performed with computations compared to experiments, while still retaining excellent or acceptable accuracy. 

In recent years, a very fast method of obtaining advanced materials has been the powder technology that uses the spark plasma sintering (SPS) as one of the modern compaction methods. SPS allows for the realization of high sintering rates by uniaxial pressing accompanied by the passage of an electric current through the sample [1,2,3]. This results in the rapid heating of the sample and, moreover, the discharges between powder particles can cause the local welding of the particles and thus contribute to even more rapid sintering [3,4,5,6,7]. These experimental improvements together with smart alloying set a suitable base for optimized NiTi-based shape memory alloys. 

Aside from the shape memory effect, NiTi-based materials also reveal superelastic behavior [1,8,9,10,11]. Both the shape memory effect and superelasticity are related to the martensitic, diffusionless first-order transformation between cubic B2 austenite (A) and martensite (M) [8,9]. In NiTi-based materials, the martensitic transformation appears in a temperature domain between −150 °C and 200 °C. The martensite phase can accept two stable crystal structures, monoclinic B19′ or orthorhombic B19 [9,10,11,12]. These two phases differ in terms of Young’s modulus, thermal conductivity, resistivity and thermal expansion coefficient [8,11], and their respective appearances thus have relevant implications on the mechanical performance of shape memory alloys (SMAs). 

The addition of a third element to stoichiometrically balanced NiTi SMAs widens the possibilities for adapting the material toward the specific needs of different applications. For instance, the Cu alloying of NiTi SMA is used to improve mechanical properties such as a high tolerance against cyclic loading or for the modification of the hysteresis of martensitic transformation (MT) temperatures [1,11,12,13]. This material is particularly used at low temperatures in biomedical and aerospace applications. The partial replacement of Ti, Ni or both by third element may be realized. The respective alloying strategy will indeed influence the phase transformation temperatures and the transition behavior between the B2→B19′→B19 sequence and will also have an effect on hysteresis, strength, ductility, shape memory characteristics and intermetallic precipitates [9,13,14,15]. Ni_2_Ti/(Ni, Cu)_2_Ti, NiTi_2_/(Ni, Cu)Ti_2_ and (Ni, Cu)_3_Ti phases were identified by X-ray diffraction (XRD) or transmission electron microscopy when Ti or Ni was replaced by Cu [1,9,13,14]. Moreover, Cu is an interesting alloying candidate in terms of the properties of amorphous SMA. Ti–Cu-based amorphous alloys are known for their high specific strength and represent promising materials for structural and functional uses, especially in biomedical applications [1,16]. Also, Neves et al. [14] showed that, during the mechanical alloying (MA) process, great amounts of vacancies, dislocations and associated lattice strain were introduced into NiTi materials. Interestingly, the treated powders presented an amorphous structure, supposedly favoring the rearrangement of Ti and Ni atoms to form small clusters. These distributed clusters may act as nucleation sites, which start to grow during tempering at higher temperatures. In this process, the high kinetic energy of balls causes a crushing of particles leading to the reduction in the particle size and local welding of particles by plastic deformation. The associated friction forces act together with diffusion, thus accelerating structure refinement and the formation of the B2 solid solution [13,14,15]. 

Optimizing the sintering and heat treatment conditions for ternary SMAs has recently been supported by Calphad computational thermodynamics and Calphad-based kinetic simulation [17]. Povoden-Karadeniz et al. [17] established a thermodynamic Ti-Ni-Cu SMA database, containing the stable and metastable phases of this system. In a computational thermokinetic evaluation of temperature- and aging time-dependent precipitation in Cu-alloyed Ti-Ni SMA, the assessed thermodynamics are combined with kinetic models for diffusion, nucleation and growth of precipitates, as implemented in the solid-state transformation of MatCalc software, version 6.03 [18]. 

**Figure 1:**
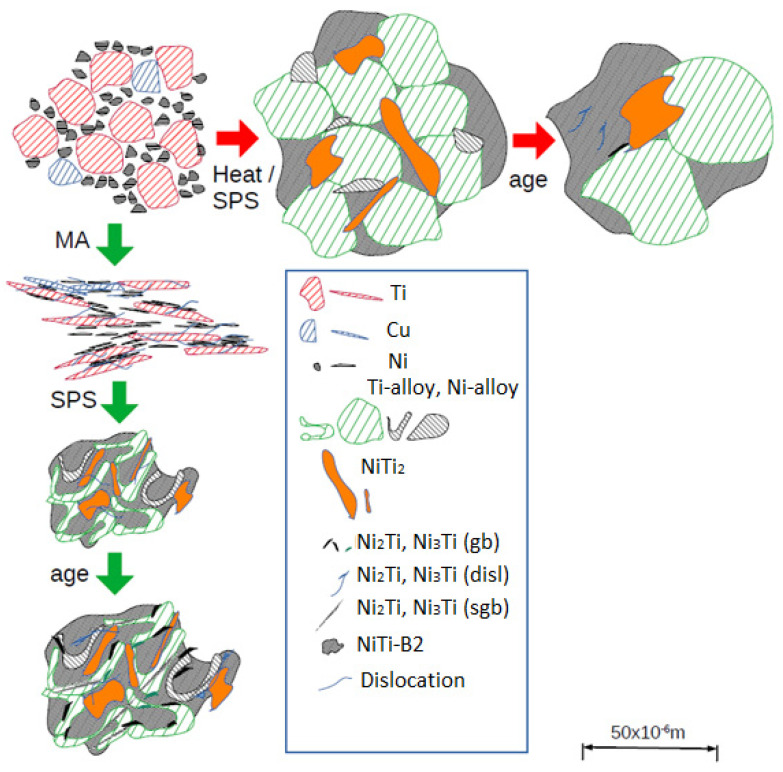
Idealized scheme of phase evolutions in different processing routes: MA-free SPS followed by aging, and MA (powder → layered/lamellar and partially cold-welded → convoluted lamellar [19]) and SPS, followed by aging [6].

Also, the sintering by spark plasma combined with heat treatment offers the possibility to obtain materials’ bulk outside thermodynamic equilibrium, enabling the formation of metastable phases in the microstructure or partial amorphous structure. Cirstea et al. [1,3,6] investigated the influence of the microstructure of Ni_45_Ti_50_Cu_5_ and Ni_40_Ti_50_Cu_10_ with MA and without MA in SPS-processed material on the mechanical properties. From the analysis of the results obtained from XRD and EDX measurements, the existence of austenite and martensite phases, Ni or Ti phases and precipitates was observed. After aging treatment, the Ni or Ti phases disappeared. The EDX and XRD showed the presence of the minority austenite phase and martensite as the majority phase. Moreover, they have confirmed that Ni_2_Ti/(Ni, Cu)_2_Ti, NiTi_2_/(Ni, Cu)Ti_2_ and Ni_3_Ti/(Ni, Cu)_3_Ti phases exist, that the samples were properly compacted and the density calculated by Archimedes’ method was 5.041 ÷ 6.664 g/cm^3^ [6]. The densification of NiTiCu materials is higher in the high-Cu samples’ content. Compared to the Ti_50_Ni_45_Cu_5_, Ti_50_Ni_40_Cu_10_ and Ti_50_Ni_40_Cu_10_-MA samples, the Ti_50_Ni_45_Cu_5_-MA sample showed less densification. In principle, the mechanically alloyed samples (Ti_50_Ni_45_Cu_5_-MA and Ti_50_Ni_40_Cu_10_-MA) are more homogeneous compared to Ti_50_Ni_45_Cu_5_ and Ti_50_Ni_40_Cu_10_, because all elements, Ti, Ni and Cu, react earlier, thus accelerating the element redistributions. The multi-precipitate phases improved the superelasticity of samples and the elastic modulus was between 71 and 82 GPa for Ni_45_Ti_50_Cu_5_ and 70 and 94 GPa for Ni_40_Ti_50_Cu_10_ materials. Vickers hardness was in the range of 326 ÷ 646 [6]. The scheme from Figure 1 summarizes the complexities of precipitation and microstructures in NiTiCu materials processed by SPS, with MA and without MA. 

In actuator applications, the thermal conductivity, the thermal diffusivity and the specific heat define a material’s ability to store and transfer heat. Research aiming at estimating these important thermophysical properties of more complex SMAs, such as ternary NiTiCu, has been performed rarely [20,21], and to date, systematic knowledge about the thermal conductivity and thermal diffusivity of Cu-alloyed SMAs is not available. The specific heat can be determined by differential scanning calorimetry (DSC), and the thermal diffusivity can be determined by the flash method (FM) [22]. FM has been previously used for the measurement of martensitic transformation temperature, thermal diffusivity and thermal conductivity of SMAs [22,23]. The thermal properties are dependent on composition, phase content, homogeneity of the material/microstructure, density/porosity of the material and the processing route. Thermal diffusivities from FM and specific heat from DSC contribute to the development of a thermodynamic properties SMA database.

Taking into account the above-mentioned, the aim of this work is to continue the investigations started in [6,17] and the understanding of the processing—microstructure correlation including precipitation—and MT—thermophysical properties of NiTiCu materials through supportive computational thermodynamics. Also, the current work compiles a thermodynamic model to simulate the sintering and aging evolution of NiTiCu materials obtained by SPS with and without MA. In order to relate materials thermodynamics to observed phase contents/microstructures, correlations of XRD with thermodynamic predictions are explored.

## 2. Materials and Methods

### 2.1. Materials

The investigated NiTiCu samples with the compositions Ni_45_Ti_50_Cu_5_ and Ni_40_Ti_50_Cu_10_ (at.%) were made at INCDIE ICPE-CA in previous studies [3,6] by powder technology with and without MA up to 15 h. The samples were made from Ni (with 10 µm particle size), Ti (with 150 µm particle size) and Cu (with <63 µm particle size) elemental powders, acquired from Merck, Darmstadt, Germany. Four samples with different processing and alloying methods are compared: without MA and with 5 at.% Cu alloyed (Ni_45_Ti_50_Cu_5_ with notation HT-P0), without MA and with 10 at.% Cu (Ni_40_Ti_50_Cu_10_ with notation HT-S0), with MA and with 5 at.% Cu alloyed (Ni_45_Ti_50_Cu_5_ with notation HS-P1), and with MA and with 10 at.% Cu alloyed (Ni_40_Ti_50_Cu_10_ with notation HS-S1). In the case of MA powders, accurately weighed Ni, Ti and Cu powders were added to a vial-ball assembly that was placed in a planetary mono mill (Pulverisette 6, Fritsch GmbH, Idar-Oberstein, Germany). The MA was conducted at room temperature in a stainless steel vial with stainless steel balls under an argon atmosphere. The powder ratio was 7:1 at a rotation speed of 250 rpm for a milling time of 15 h. The material was sintered using HPD 25–FCT Systeme spark plasma sintering equipment, GmbH, Rauenstein, Germany. The desired amount of alloy powder was placed in a graphite die between graphite foils. Once the graphite die containing the alloyed powder was introduced into the sintering furnace, it was pressed in vacuum at a pressure of 50 MPa throughout the sintering cycle to establish electrical and thermal contact, at a sintering temperature of 900 °C and holding time of 5 min. Cylindrical-shaped samples with a 20 mm diameter and about 4–5 mm height were obtained. After ejection from the die, the samples were mechanically processed/polished to remove the graphite foils. In order to increase the alloy homogeneity, the NiTiCu samples were thermally aged in an oven in two steps as follows: temperature increasing by 100 °C/min until 850 °C, holding for 1 h, cooling by 100 °C/min until 500 °C, holding for 30 min and cooling again at room temperature by 100 °C/min in an argon atmosphere. A clean grain boundary (gb) area with direct grain-to-grain contact was obtained due to the physical activation of powder particle surfaces during pulsed current application. 

### 2.2. Methods

#### 2.2.1. Microstructural Investigation by XRD

Materials for metallographic analysis were ground with silicon carbide paper from 600 up to 2400 grit size. The conditions of applying XRD measurements and density measurements are described by C. D. Cirstea et al. [6] in some previous studies. In order to evaluate the NiTiCu-SMA crystallinity during various technological procedures, the crystallinity index (*CI*(%)) was calculated from XRD difractograms. The crystallinity index of the studied samples was performed with Origin Pro 8.5 and was calculated by the following equation [24]:

(1) $$CI(\%)=100\times \frac{S_{c}}{S_{t}}$$

where *S_c_* represents the integral area of the crystalline domain and *S_t_* represents the integral area of the total domain, respectively.

#### 2.2.2. Computational Methods

##### Thermodynamic Model for the Ni-Ti-Cu Based B2 Phase

Thermodynamic Ni-Ti-Cu databases have been established [17,25], where the reassessment of the Ni-Ti subsystem by Povoden-Karadeniz et al. [26] has focused on metastable phases of the system, simplifying the model of the austenitic B2 phase in regard to the (assumed) distribution of vacancies in the phase. Zhang et al.’s revised Calphad description of Ni-Ti-Cu [27] allowed for vacancies to sit in both sublattices of the split model for the ordering of the B2 phase, the according sublattice formula reading (Cu, Ni, Ti, Va)_0.5_(Cu, Ni, Ti, Va)_0.5_(Va)_3_. In contrast, Povoden-Karadeniz et al. [26] simplified the description of B2 (BCC_B2), still with a good reproduction of phase boundaries but without the requirement of substitutional vacancies associated with questionable compound energies, proposing the description (Cu, Ni, Ti)_0.5_(Cu, Ni, Ti)_0.5_(Va)_3_. This model was also used recently to describe phase stabilities in the Ni-Ti-V system in agreement with experiments [28] and can be used as the matrix phase in thermokinetic simulations. Alternatively, for equilibrium and metastable thermodynamic computations, we propose a 4 sublattice description (Ni)_2_(Ti)_2_(Ni)_1_(Ti, Va)_1_ for the same phase, allowing for highly Ti-defective regions in the Ti-understoichiometric Ni-Ti base SMA matrix, as proposed by first-principles analysis, the phase named “FP_B2” in the developed open-source thermodynamic database mc_sma [29]. This phase can precipitate in a thermokinetic simulation but cannot act (different from BCC_B2, see above) computationally as an alloy matrix phase. 

##### Thermokinetic Model for the Ni-Ti-Cu System

In order to relate the thermal evolution of the studied alloys with thermophysical properties, Calphad-evaluated phase stabilities in the ternary system Ni-Ti-Cu [17] are compared with XRD analyses of the samples. Thermokinetic aging simulations at 850 °C and 500 °C are performed with the solid-state transformation software MatCalc [30]. For precipitation simulation, this software takes into account the thermodynamics of the matrix phase and precipitate, particularly the chemical potential differences between the alloy matrix and precipitates, as well as interfacial energies and diffusivities, which are required for solving nucleation and growth equations. Tabulated data of the diffusivities of alloyed atoms in the matrix phase are obtained by the diffusion database mc_sma.ddb [29]. The effect of the chemical ordering of a B2 matrix on the diffusivities is considered by the approach of Helander and Agren [31]. Typically, interfacial energies are evaluated by the generalized nearest-neighbor broken bond model [32]. However, here, for the special case of an ordered austenitic B2 matrix with almost balanced Ni and Ti (at.%), the model has failed to date, and we assume a constant interfacial energy of 200 mJ/m^2^ for Ni_3_Ti and 100 mJ/m^2^ for (enthalpically) metastable Ni_2_Ti, in which case nucleation theory [33] suggests a lower value. The chosen values are in the range of previously suggested interface energies in NiTi SMAs [6,34]. Precipitates are considered to form heterogeneously at grain boundaries, in agreement with previous experimental findings [35]. MA associated with partial amorphization also leads to shear bands, which—together with the recombination of locally concentrated dislocations—accumulate to form subgrains, being separated by low-angle grain boundaries (sgb). Possible precipitation at dislocations (disl) in samples without MA and with MA, competing with gb precipitation, is tested in the simulations, with an assumption of 1 × 10^12^ dislocations per m^2^, unstrained. However, excess dislocations inherited from the mechanical treatment are also rapidly annihilated at the elevated temperatures of SPS; and thus their significant effect on precipitation and thermal properties appears unlikely. This behavior is roughly mimicked by a small excess of dislocations used for kinetic simulations of MA material (5 × 10^12^ dislocations per m^2^, strained). For the kinetic modeling of the grain boundary and subgrain boundary nucleation sites (*N_gs/sg_*), the tetrakaidekahedron concept [35,36] is adopted. The tetrakaidekahedron (*tdh*) represents a plane-faced body which enables regular space filling in any polycrystalline material. Its volume, *V_tdh_*, surface area, *S_tdh_*, and radius are unambiguously defined [36]. This allows us to define the number of nucleation sites [37],

(2) $$N_{gs/sg}=\frac{S_{tdh}}{2V_{tdh}}{\left(\frac{N_{A}}{V_{m}}\right)}^{2/3}$$

*N_A_* in Equation (2) is the Avogadro number, and *V_m_* is the molar volume.

#### 2.2.3. Thermophysical Investigations 

##### DSC Measurements

The MT temperatures are determined by DSC via a model 204 F1, Netzsch Differential Scanning Calorimeter (GmbH, Selb, Germany) operating in the temperature range of −70/+130 °C at a cooling/heating rate of 10 °C/min in a nitrogen protective atmosphere. The start and finish temperatures for the direct martensite phase (M_s_, M_f_) and reverse austenite phase (A_s_, A_f_) transformations are determined from the DSC thermograms by the tangential method using PROTEUS software, https://analyzing-testing.netzsch.com/en/products/software/proteus, 32 bit, MS-Windows [38] and Origin Pro 8.5. The preparation of samples for analysis was carried out in accordance with the ASTM 2004 standard [38] and for this study, an electro-discharge machine was used to cut the samples.

##### LFA Measurements

Cylindrical samples were tested for thermal conductivity and thermal diffusivity using a Netzsch LFA 457 laser flash analyzer, Geratebau GmbH, Selb, Germany. The samples are 12.7 mm in diameter with a thickness of 2.51 mm. The surface of the NiTiCu samples is coated with a graphite solution to facilitate fast absorption of the flashlight. The LFA measurement was performed in isothermal steps from 25 °C up to 175 °C, with an increment of 25 °C. Five shots were taken at each temperature, and the measured conductivity and diffusivity results were averaged, accordingly. The thermal diffusivity, *α*, and the thermal conductivity, *k*, are obtained employing the Parker flash method in accordance with the ASTM E-1461 standard [39]. The LFA 457 equipment allowed for the direct measurement of the thermal diffusivity, while the specific heat of the materials was determined by a differential method, using a reference sample. The Cowan model with linear baseline correction was used for the analysis of the data. The analysis included the possibility of heat loss from both the front and back faces of the sample. For the specific heat measurement, the magnitude of the temperature rise of an unknown sample was compared to that of the Inconel standard.

## 3. Results

### 3.1. XRD Investigation of Microstructure and Phase Stabilities 

**Figure 2:**
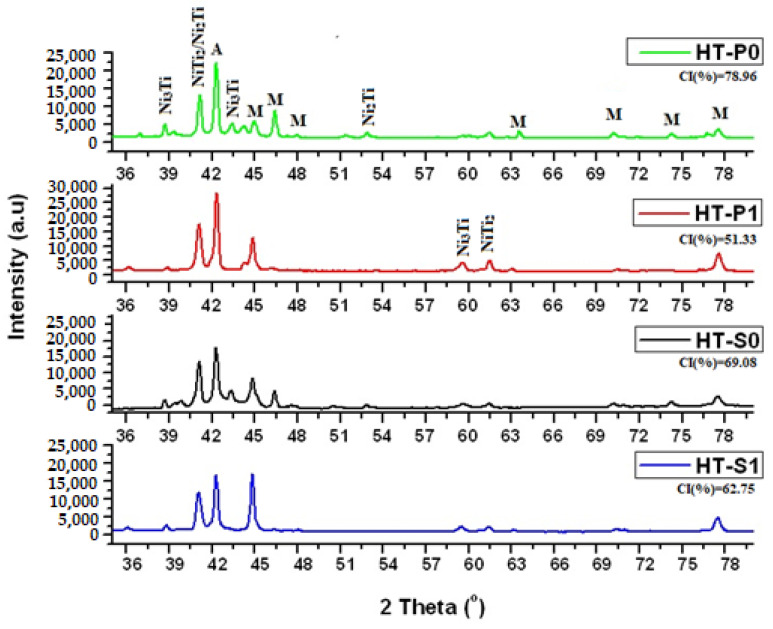
X-ray diffraction patterns of the NiTiCu materials correlated.

**Figure 3:**
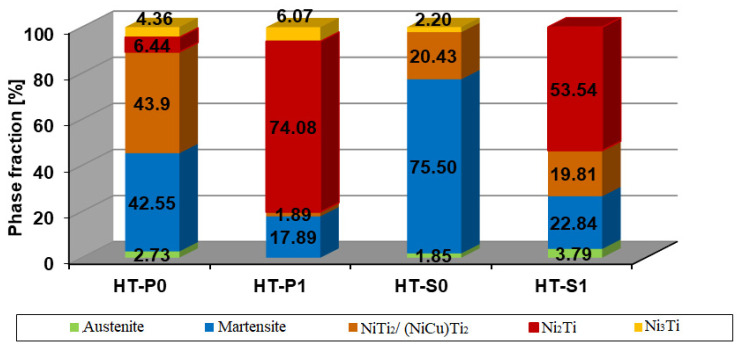
Experimental phase fractions of NiTiCu materials from [6].

The XRD patterns are shown in Figure 2 and include the CI values. Figure 3 is correlated with the phase fractions obtained by Cirstea et al. [6] from XRD measurements for the NiTiCu materials after the respective manufacturing processes.

Fast heat transfer from a high temperature (900 °C) and aging steps at 850 °C and 500 °C promoted monoclinic martensite, together with Ni_3_Ti, NiTi_2_/(CuNi)Ti_2_ and Ni_2_Ti. These phases always contained considerable amount (a few at.%) of Cu. The Cu-containing Ni-Ti precipitates appear more frequently in alloys manufactured by SPS, as compared with casting technologies [10,16], which supports the high importance of kinetics for their formation.

In Figure 3 represents the phases as identified by X-ray analysis from Cirstea et al. [6] of comparable regions within the studied samples. However, there is no indication of higher Cu contents in any analyzed Ni-Ti phases, in a concentration range that would match the high Cu solubility in ternary equilibrium phase tau1 of the Ni-Ti-Cu system, with approximately equal atomic fractions of Ni, Ti and Cu [40]. Tetragonal tau2, around the atomic stoichiometry Ni_56_Ti_40_Cu_4_, was also not found experimentally. In contrast, the occurring intermetallic precipitates represent very likely solutions based on binary Ni-Ti phases, which are able to dissolve several at.% of Cu [6]. The following intermetallic phases and their crystal structures have been securely identified: Ni_2_Ti/Ni_2_(Ti, Cu) (hexagonal, space group R-3m), Ni_3_Ti/Ni_3_(Ti, Cu) (hexagonal, space group P63/mmc) and NiTi_2_/(Ni, Cu)Ti_2_ (cubic, space group FD-3m). It should be noted that for observed Ni_2_Ti, the Calphad Ni-Ti phase stability required reassessment, since, with the previous thermodynamic description, the phase can under no circumstances be stabilized. This problem is solved when the enthalpy of the formation of the phase, in agreement with ab initio analyses [25], is shifted to more negative values, closer towards the theoretic enthalpies of the formation of other Ni-Ti intermetallics. Moreover, Cu was allowed to dissolve in the phase in the thermodynamic re-parametrization. Cu has a notable effect on the thermodynamic stabilization of the phase.

It is evident that the phase content in the observed microstructures does not reflect equilibrium, but it in contrast, it strongly represents non-equilibrium and—microstructurally—considerable spatial fluctuations in element concentrations. Further, equilibrium would actually contain (even the state with austenitic B2-phase rejected) fewer phases than the observed phase contents, both after sintering and aging. In the HT-P0 and HT-S0 samples, the martensite (mixed B19′ + B19) and (Ni, Cu)Ti_2_ phase predominate, while in the HT-P1 and HT-S1 samples, a small amount of martensite and a large amount of Ni_2_(Ti, Cu) precipitate were identified. The amount of (Ni, Cu)Ti_2_ can be reduced by using high heating rates in the processing, like in the case of SPS. Further, mechanical alloying before sintering in SPS and heat treatment at temperatures ranging from 400 to 900 °C are preferable [26,27]. Cirstea et al. [6] confirmed that the phase fraction of (Ni, Cu)Ti_2_ was reduced after mechanical alloying in HT-P1 and HT-S1, as compared to HT-P0 and HT-S0. 

### 3.2. Thermodynamics and Thermokinetic Analysis Validated by Experiments

#### 3.2.1. Thermodynamic Equilibrium Computation of Ni-Ti-Cu System

- Metastability of Ni-Ti-Cu Base Intermetallics

**Figure 4:**
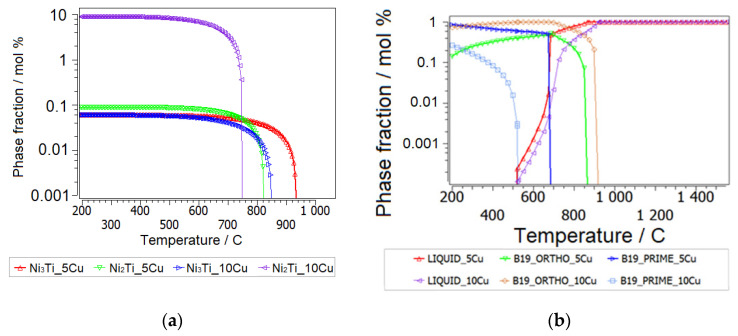
Isothermal ternary section of the Ni-Ti-Cu phase diagram at 900 °C, metastable phase fractions in Ni_51.5−x_Ti_48.5_Cu_x_ (x = 5, 10) rejecting ternary tau-phases (**a**), and metastable B19–B19′ phase relations in Ni_51.5−x_Ti_48.5_Cu_x_ (x = 5, 10) (**b**).

Computational thermodynamics employing the database mc_SMA.tdb delivers important information of the dissolution temperatures of stable and—rejecting equilibrium phases from the computation—also the theoretic dissolution temperatures of metastable phases, as depicted in Figure 4a. These temperatures represent the maximum temperature of any (also kinetically driven) occurrence of a respective phase. The metastable solvi of (Ni, Cu)_3_Ti and (Ni, Cu)_2_Ti (or tau 1) lie consistently below 900 °C, even at *X*(Ti) = 0.48, but have been observed in the microstructure in as-sintered samples. The according metastable phase fractions plot (see Figure 4a), assuming non-equilibrium, and rejecting the tau-phases in the computation, tells us the relative stabilities of the intermetallic phases for nonstoichiometric Ni_51.5−x_Ti_48.5_Cu_x_ (x = 5, 10) as a function of temperature between 25 °C and beyond 900 °C, thus covering the temperatures of the aging treatment, thermal properties investigation and the sintering temperature. 

The metastable results (tau-phases rejected from the simulation) obtained by thermodynamic simulation for off-balanced (Ni, Cu):Ti are in qualitative accordance with the XRD measured phases presented in [6] and summarized in Figure 2 and Figure 3 for HT-P0 and HT-S0.

Considering equilibrium and balanced (Ni, Cu):Ti (at.%), both studied compositions would show a B2 single phase. However, if during a short time and high sintering rate with SPS, equilibrium single-phase austenite is not obtained and even slight fluctuations in Ti around its nominal concentration occur, the metastable microstructure at elevated temperatures can reveal a complex phase assembly of NiTi-based solid solutions with the Cu of the formulas (Ni, Cu)_3_Ti and (Ni, Cu)_2_Ti at Ti-understoichiometry relative to balanced (Ni, Cu):Ti (at.%) and (Ni, Cu)Ti_2_ at Ti-overstoichiometry. Note that even with some Ti deviations from the nominal concentration, the austenitic B2 phase and (Ni, Cu)Ti_2_ are predicted to be the only stable phases at the sintering temperature of 900 °C, in case the ternary tau-phases are not formed (they have not been observed experimentally). An explanation of a deviating matrix composition from the initially adjusted one by powder metallurgy takes into account the effect of a large primary NiTi_2_ phase fraction (here, the phases are denoted as “primary” when they evolve due to SPS, different to secondary precipitates which form during subsequent aging), observed already in the HT-P0 and HT-S0 conditions (without aging) and strongly exceeding the thermodynamically calculated (approx. 3 at.% of the phase at 900 °C) phase fraction. This was also observed by Novak et al. [13] in SPS-processed NiTi SMAs and is further discussed in Section 3.3.1. When the metastable situation of a large amount of NiTi_2_ is allowed to interact with a defective NiTi B2 matrix (the FP_B2 phase mentioned above), the latter phase can become Ti-understoichiometric, with around 48.5 at.% Ti.

- Metastability of Ni-Ti-Cu Base Martensite Phases

Our multi-component SMA database includes the low-temperature martensitic phases B19 and B19′. These phases have been described in the pure Ni-Ti system by Tang [41]; however, their description required some re-parametrization in order to allow for combination with the Ni-Ti assessment by Povoden-Karadeniz et al. [38] and the extension to Ni-Ti-Cu, which has been discussed in [17]. After rejecting the austenitic B2 phase and intermetallic phases from the computation, we may interpret the relative metastability of orthorhombic B19 versus monoclinic B19′ and their changes with increasing Cu alloying and balanced (Ni, Cu):Ti, as seen in Figure 4b. The computation clearly shows the predominance of B19′ over B19 at 5 at.% of Cu alloying and the opposite at a higher Cu alloying of 10 at.% Cu. B19 becomes enthalpically slightly stabilized relative to B19′ as a function of increased Cu-alloying. For 5 at.% Cu alloying, we calculate (in J/mol atoms) the enthalpy of formation of B19 phase *H*_f_(B19) = −34,204 at 298.15 K and the enthalpy of formation of B19′ phase *H*_f_(B19′) = −35,345, at 298.15 K. In the case of *x*(Cu) = 0.15, enthalpy of formation of B19 phase *H*_f_(B19) = −28,941 at 298.15 K and enthalpy of formation of B19′ phase *H*_f_(B19′) = −28,650 at 298.15 K. This stability change (more negative enthalpies indicate higher stability) is confirmed by X. Yang et al.’s [5] ab initio formation energies of B19′ and B19 phases. Relative to NiTi-B2 metastable NiTi-B19′ and NiTi-B19 are predominant (matrix) phases, with the former being particularly relevant at low temperatures up to 400 °C, prior to the Cu-containing Ni-Ti solid solution. Whereas the metastable formation and decomposition temperatures of the intermetallic Cu-containing Ni-Ti solid solutions remain relatively unchanged between 5 and 10 at.% of Cu alloying, the metastable dissolution temperature and phase fraction of orthorhombic NiTi-B19 increases considerably towards increased Cu alloying. In other words, Cu strongly stabilizes NiTi-B19. However, the relative enrichment of intermetallic phases over matrix phases in the material with *lower* Cu alloying and vice versa at higher Cu alloying (see Figure 3: XRD-analyzed phase fractions after aging) without MA cannot be reproduced by the thermodynamic computation, and strong additional kinetic constraints are thus expected to govern this behavior. This is discussed in the next section. This is also confirmed by the fact that sintering employing *fast* SPS led to a *higher* fraction of NiTi_2_ and Ni_2_Ti precipitates, which is in accordance with Ye et al. [19]. Diffusivities in the Ni-Ti martensitic phases lack any experimental background, and these phases can thus not be used as an alternative matrix phase in kinetic simulations to date.

**Figure 5:**
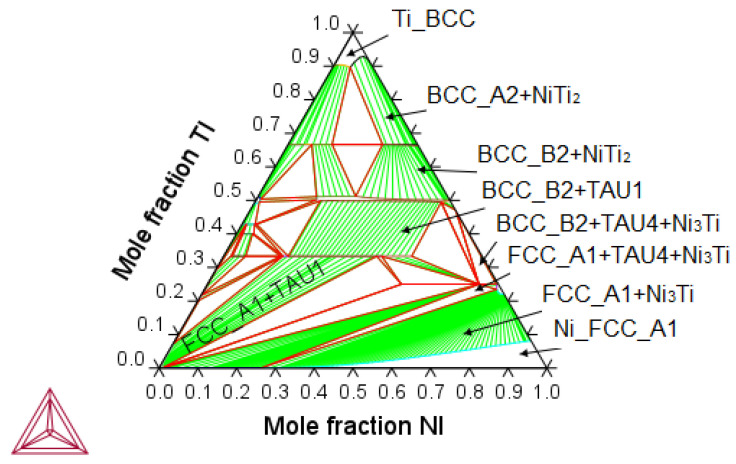
Isothermal ternary section of the Ni-Ti-Cu phase diagram at 900 °C. Equilibria on the Ni-Ti rich side.

The calculated isothermal Ni-Ti-Cu equilibrium phase diagram represents the system at sintering conditions, at 900 °C (Figure 5). It is seen that Ni_2_Ti is a metastable phase, relative to Ni_3_Ti (the former one does not appear in the equilibrium phase diagram).

#### 3.2.2. Thermokinetic Precipitation and Phase Transformation Simulation 

The multi-component SMA database of this study, containing the elements Ni-Ti-Cu-V, is presented in paper [25,40]. In order to allow for kinetic simulations for second-phase precipitation in B2-austenite, diffusivities of the ordered phase have been parametrized [24].

We started our simulations with a default setup in terms of possible metastable heterogeneous precipitate phases, which involved at the beginning also Ni_2_Ti_3_ and Ni_3_Ti_4_. However, these phases have not been observed experimentally in the studied material, and we thus decided to reject them from the simulation. A final judgement on the role of these precipitates will indeed need some further experimental high-resolution analysis, in terms of chemical and crystal structure.

- Sintering

**Figure 6:**
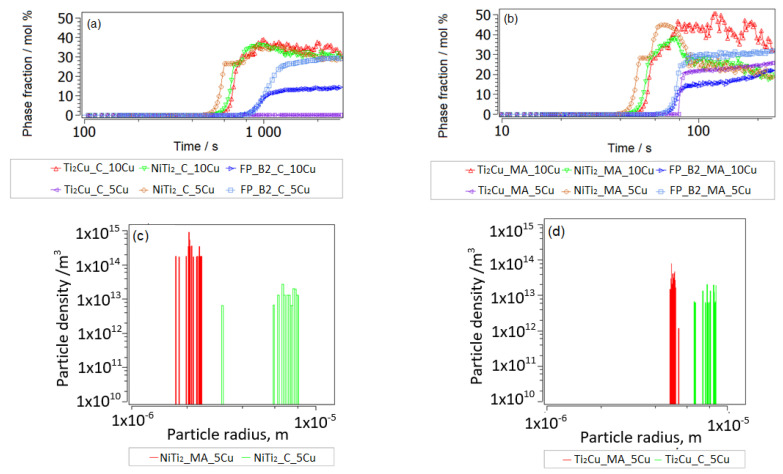
Metastable precipitation of NiTi_2_ and Ti_2_Cu and transformation of NiTi_2_ to FP_B2 in Ti-rich solution after MA and fast heating, followed by 150 s at 900 °C and 5% Cu/10% Cu in NiTiCu. Evolving phases are denoted by “MA” (**a**) and phase evolution during conventional heat treatment and sintering conditions. Evolving phases are denoted by “C” (**b**). (**c**,**d**) Particle distributions are shown for MA and SPS and conventional treatment at 10% Cu with exemplary Cu alloying (the comparison is similar for NiTi_2_ with 5% Cu, but Ti_2_Cu after “C” processing is missing). Fluctuations of simulated phase fractions for 10% Cu have numerical reasons and are not to be interpreted.

Ti-depletion or Ti-enrichment is particularly related to the sintering in powder metallurgy, as it has been described for NiTi [42] as follows. Instead of the thermodynamically straight-forward mixing of the same amounts of Ni and Ti to form the NiTi-B2 phase, Ti-bcc powder prefers to react with (relatively fast diffusing) Ni in appropriate stoichiometric mixing, forming NiTi_2_, which then can transform (in part) into NiTi-B2. Remaining Ni-rich powder regions (with Ti diffusion being too slow to reach equilibrium) can react with Ti to form Ni_3_Ti and Ni_2_Ti. The considered reactions are clearly off pure equilibrium reactions and are shown in Figure 4a for typical fast heating rates (10 °C/s) after MA, followed by short (150 s) isothermal treatment at 900 °C, representing the SPS process. We assume a Ti-Ni-bcc solution matrix with 5 at.% Cu (Figure 6a) and 10 at.% Cu (Figure 6b). Equilibrium calculations show that Ti_2_Cu is actually an equilibrium phase under these conditions, and it is considered also in the thermokinetic precipitation simulation. We also show the typical heat treatment (heating rate 1 °C/s) for conventional sintering (approx. 1 h) at the same temperature.

Ni_3_Ti and NiTi_2_ phases are considered to nucleate at the surface (triple-junctions) of Ti and Ni particles, respectively, and for FP_B2, direct particle transformation modeling in MatCalc is employed. Moreover, Cu-Ti particles are defined (see Figure 1).

**Figure 7:**
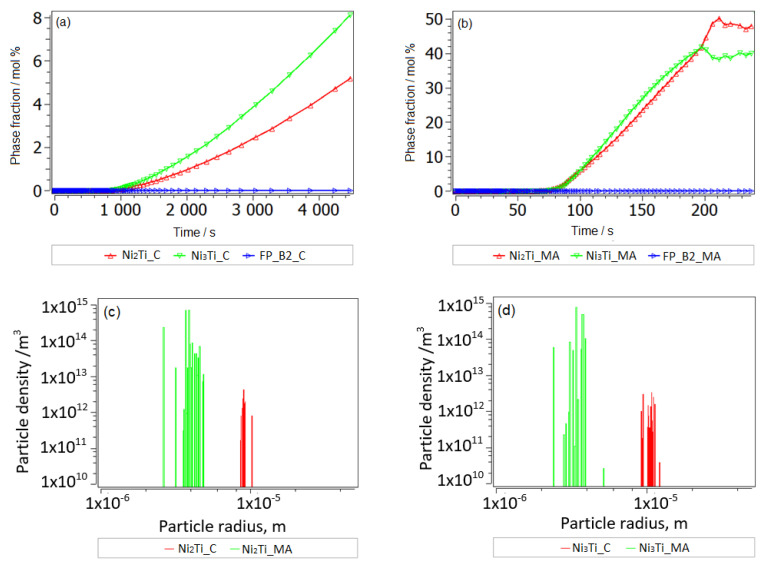
Phase evolution at slow heating to conventional sintering (“C”) (1 h, 900 °C) in Ni-rich fcc solution (**a**), and analogous for the MA and SPS (“MA”) process (**b**). Exemplary for 5% Cu. Particle distributions are shown in (**c**,**d**).

Figure 7 shows the phase evolution in a Ni-rich fcc solution due to conventional treatment, Figure 7a, and during SPS after MA, Figure 7b. The Ni-rich intermetallic phase formation is considerably promoted by the fast rates and small grain sizes in MA and SPS. Only the case of 5 wt.% of Cu alloying is presented, since Cu-rich precipitates do not form in this case, and the influence of increasing Cu to 10 at.% for these phase formations has been shown to be negligible. 

The following results of these phase nucleations and transformation (NiTi_2_ → FP_B2) simulations are particularly relevant:

(1) Simulated precipitate radii are consistently about a half to one order of magnitude smaller in MA and SPS, as compared to conventional heat treatment, and the opposite is true for particle number densities. This trend shows the dominating role of decreasing grain sizes due to MA and SPS for the precipitate evolution. 

(2) Ni_3_Ti (and a small fraction of Ni_2_Ti) largely forms in MA and SPS Ni-rich fcc solution. This agrees with the experimental trend (see Figure 3). 

(3) Only NiTi_2_ in Ti-rich sample regions transforms to FP_B2. The FP_B2 resulting from this phase transformation reveals Ti-understoichiometry, with x(Ti) = 0.485. For precipitation during aging after sintering, this is relevant, since only off-stoichometric BCC_B2 is expected to lead to further excess precipitate fractions of secondary Ni-Ti particles.

(4) MA and SPS indeed led to lower NiTi_2_ precipitation than during conventional heat treatment, in agreement with the results from [6]. 

(5) The relative fraction of the Ti_2_Cu phase is predicted to be higher with MA and SPS. Cu-Ti particles play a more pronounced role in the Ti-rich solid solution matrix when higher Cu alloying is added. In fact, the predicted competition between a Cu solution in binary Ni-Ti intermetallics and the formation of a Ti-Cu phase is not completely sure to date and must be confirmed by further refined experiments.

- Aging

For the time being, mean-field thermokinetic simulation with MatCalc [37] allows for the definition of the austenitic B2 phase as a matrix phase and the test of nucleation and the growth of secondary precipitates. The parameter setup for the kinetic precipitation simulations is summarized in Table 1. 

**Figure 8:**
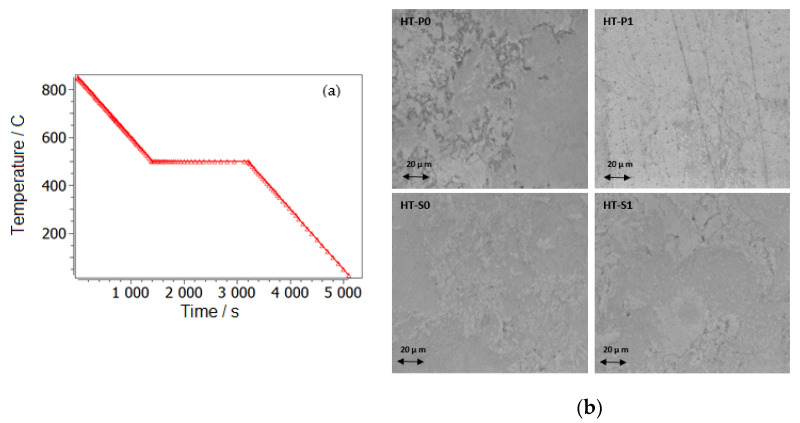
The heat treatment considered in all aging simulations, starting with cooling from the 850 °C aging step (**a**); SEM observation from MA and SPS and aged NiTiCu materials (**b**).

**Figure 9:**
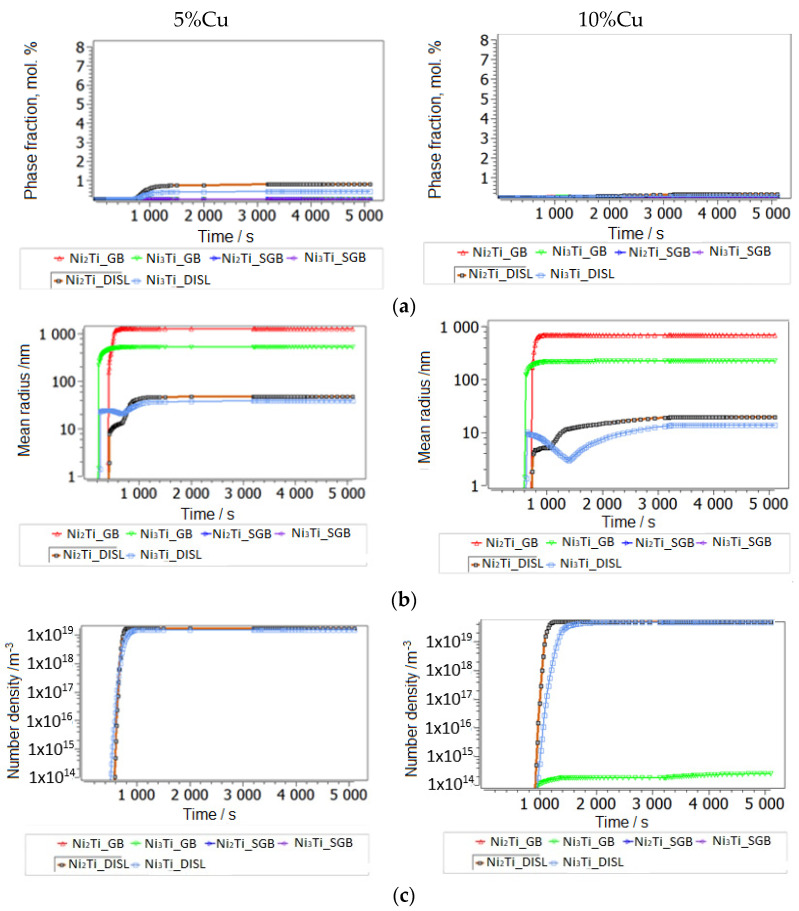
Secondary precipitation after aging at steps 850 °C and 500 °C in NiTiCu without MA; precipitate fractions (**a**), radii (**b**) and number densities (**c**). In each of the plots, the phase of the largest fraction is marked.

**Figure 10:**
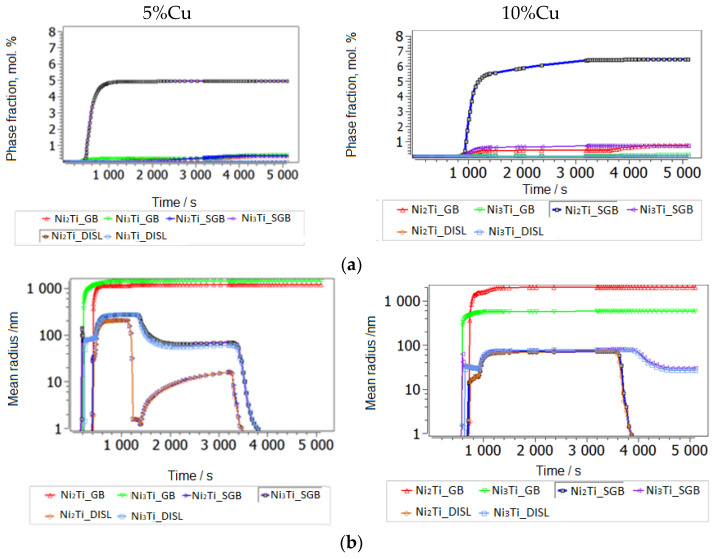

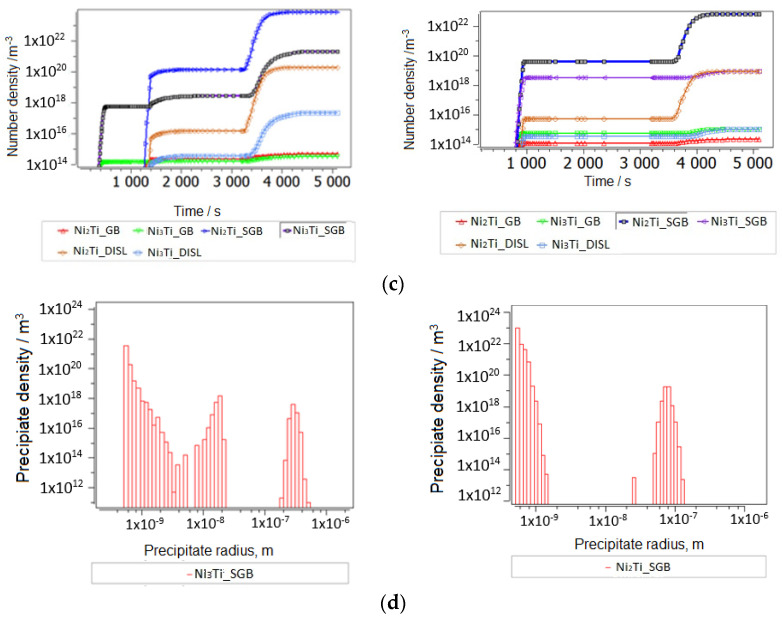
Secondary precipitation after aging in steps at 850 °C and 500 °C in NiTiCu, in MA and SPS samples; precipitate fractions (**a**), radii (**b**), number densities (**c**), particle distribution (**d**). In each of the plots, the phase of the largest fraction is marked. The tiny particles (approx. 1 nm radius) in the bimodal precipitate distribution (**d**) form during the last cooling step after aging, below 500 °C.

We propose a mean diffusion increase by a factor of 10 (MA and SPS) in the matrix, which shall imitate partial amorphization [42,43]. With such an identical setup for the cases of lower and higher Cu alloying, we have reproduced the experimental observation of preferred second-phase precipitates in a Ti-understoichiometric B2 matrix, Ni_51.5−x_Ti_48.5_Cu_x_ (x = 5, 10), shown in Figure 8, Figure 9 and Figure 10. Secondary Ti-Cu precipitates do not form in the aging simulations, and they are thus not considered. In Figure 8a is the heat treatment considered in all aging simulations, starting with cooling from the 850 °C aging step and SEM images. The SEM observations of the NiTiCu materials are shown in Figure 8b and are correlated with EDS measurements from Cirstea et al. [6].

It is seen that the surface of the materials was compact and mostly demonstrated a good densification as also confirmed by the density data of Cirstea et al. [6]. 

Some porosity is present locally at grain boundaries, especially in Ni_45_Ti_50_Cu_5_. The densification of NiTiCu materials is higher in the high-Cu samples due to increased diffusion in NiTi as a function of alloyed Cu [4,43]. Compared to the HT-P0, HT-S0 and HT-S1 samples, HT-P1 showed less densification. In principle, the MA samples (HT-P1 and HT-S1) are more homogeneous compared to the ones without MA (HT-P0 and HT-S0), because all elements, Ti, Ni and Cu, react earlier with MA; thus, the element redistributions are accelerated.

The simulation starting with cooling from the first high-T aging step at 850 °C is shown. This temperature is sufficiently high to remain in the pure Austenite (BCC_B2) matrix phase without secondary precipitation. Aging-processed alloys have a larger grain size of 50 microns without MA (Figure 9a–c). Simulation results for grain-refined samples (MA) down to around 10 microns are shown in Figure 10a–d. 

A grain size of 10 microns shall represent the MA and heat-treated processing case. Indeed, a strong influence of grain size on precipitation prevails for grain boundary precipitates (second-phase Ni-Ti-based particles are found at grain boundaries in SMAs [34]). In addition to grain refinement by MA, clearly some amorphization has been confirmed by XRD, whereby typical amorphous/amorphous-crystalline nanolamellae have been reported [19,44]. Lamellae boundaries are comparable to low-angle grain boundaries, which in MatCalc are represented by the precipitation sites of subgrain boundaries. These additional sites, with sizes around 100 nm, were also considered in the kinetic precipitation simulation of MA samples. Lamellar heterogeneous regions are also typical to the cold-welding of mechanically alloyed elements [45,46,47,48], which is due to the low temperature of the process being preferred over their thermodynamic mixing or formation of the intermetallic phase. Towards elevated temperatures, when subsequent processes are fast (SPS), part of the inherited non-equilibrium convoluted lamellae likely remain in the microstructure, providing heterogeneous low-angle boundaries for precipitation. The last type of potential, energetically preferable sites for the nucleation of precipitates are the dislocations. At the end of 8 h of MA NiTiCu SMA, the X-ray analysis evaluation [47] yielded a very high dislocation density of 1 × 10^16^ dislocations per m^2^, which is actually close to the typical values for fresh martensitic steel and comparable to the results of metallic MA material [48]. In order to use reasonable dislocation densities after the subsequent heating to thermal sintering and aging conditions, with a lack of precise experimental quantifications, we employ a rough estimate of annealing dislocations during heating by using the relation between the equilibrium dislocation density of B2 set to 5 × 10^11^ m/m^3^ dislocations and an activation energy term for dislocations’ annihilation, assumed to be −23 kJ, using simple Equation (2), resulting in a realistic annihilated dislocation density after heating to the sintering temperature of 900 °C of around 5 × 10^12^ m/m^3^. This relatively low dislocation density is associated with negligible precipitation at dislocations, compared to the other sites mentioned.

In all materials, the most important secondary precipitation simulation results, presented in Figure 9 and Figure 10, reproduce experimental trends when Ti understoichiometry inherited from SPS is allowed for the BCC_B2 matrix. Moreover, the heterogeneous precipitation of Ni-Ti intermetallics is enhanced with MA. Towards increased Cu, Ni_3_Ti particles become less dominant, whereas Ni_2_Ti becomes more relevant. Since the fractions of primary versus secondary precipitate fractions are not separated experimentally to date, the predicted trend of preferred Ni_2_Ti precipitation at higher Cu alloying cannot be validated. We also cannot define the (partial) replacement of the austenitic B2 matrix by B19 and B19′ due to martensitic transformation and the possible influences on the evolution of intermetallic Ni-Ti phases. Figure 1 and Figure 3 indicate the complex inter-relations between the increasing/decreasing martensitic phase fraction and changing Ni-Ti intermetallic particle fractions as a function of processing and Cu alloying. Further, simulative consideration of the R phase is not possible to date, since the role of Cu on its stability and Cu solid solubility in the phase is unknown. 

An increasing influence of precipitates on thermal properties is related to their characteristic distributions at grain boundaries and other interfaces and their number density and size, since these properties are directly related to the change in the number of interfaces and the complexity of the interface–precipitation or dislocations–precipitation interrelations in the microstructure. 

### 3.3. Thermophysical Properties Results

#### 3.3.1. DSC Results and Interpretations of Transformation Characteristics

The measurements confirm that both alloy compositions undergo a single-step transition between the A and M phases, with MT temperatures below 100 °C. The measured MT temperatures are listed in Table 2. 

**Figure 11:**
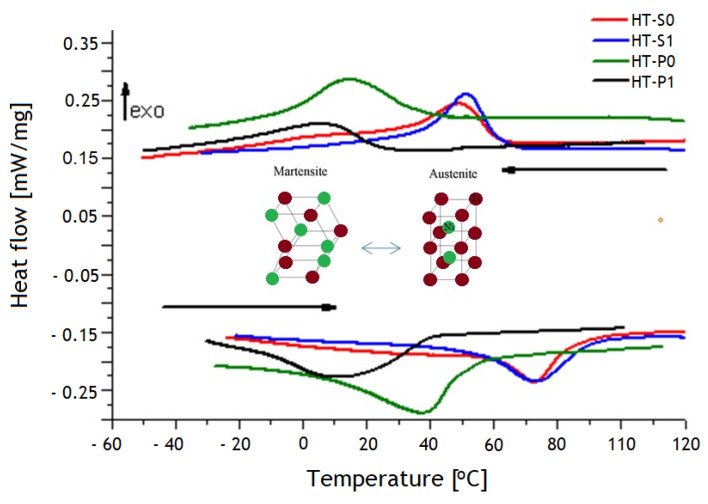
DSC curves of NiTiCu alloys. Upper diagram—cooling curves, lower diagram—heating curves in correlation with crystallographic structure from austenite and martensite.

Figure 11 shows the DSC curves of all NiTiCu samples. Enthalpies of the reversible austenite (A) to martensite (M) transformation for all alloys are approximately 5 J/g. 

The experimental phase fractions of Cirstea et al. [6], summarized in Figure 2 and Figure 3, indicate that the nature of DSC confirmed that the transformation of A↔M (or MT) is B2↔B19′, except for lower-Cu-containing HT-P0 and HT-P1, where the transformation from austenite to martensite also involves B19, thus being of type B2↔B19↔B19′. Note that the DSC experiments do not allow for separating the associated peaks of such a two-step transformation. The obtained trends from DSC are in agreement with results from Nam et al. [15] who showed that only the B19′ martensite is formed for NiTiCu alloys obtained by MA and SPS at 850 °C with less than 10 at.% Cu content, whereas only the B19 martensite is formed in those alloys with a Cu content above 10 at.%. The same results were obtained by Kang et al. [49] and Goryczka et al. [9,50] when investigating the microstructure and transformation behavior of NiTiCu shape memory alloy powders fabricated by mechanical alloying with a rotating speed of 250 or 350 rpm in the range 5–15 at.% Cu. Our DSC results further show that the effect of MA on MT is particularly pronounced with lower Cu alloying, whereas the effect is small with higher Cu alloying. This goes along with the microstructural observation of a considerable increase in the martensite fraction in HT-S1 as compared to HT-S0, whereas the martensite fractions in HT-P0 and HT-P1 are much closer to each other. 

Regarding the M↔A transformation, it can also be noticed from DSC curves (Figure 11) that the HT-S0 and HT-S1 samples exhibit almost an overlapped peak, whereas the HT-P0 and HT-P1 samples do not. This happens due to the partial crystalline structure. In the case of Ni_40_Ti_50_Cu_10_, both HT-S0 and HT-S1 have very similar values for CI, compared with HT-P0 and HT-P1. Taking into account the results from Cirstea et al. [6], the porosity of the Ni_40_Ti_50_Cu_10_ alloy, both the HT-S0 and HT-S1 samples, has similar values (3.29% and 2.72%), while in the case of Ni_45_Ti_50_Cu_5_, the porosity of the HT-P1 sample is almost double in relation to the HT-P0 sample (18.73% vs. 9.19%). These results are in accordance with Goryczka et al. [50]. They observed a similar MT behavior, but the duration of mechanical alloying applied to obtain NiTiCu materials was more than 20 h. 

The metastability thermodynamic simulations shown in Figure 4 confirm that the thermodynamic stability of martensite depends on the amount of Cu alloying and a composition-dependence of the *T*_0_ temperature is thus also expected. *T*_0_ represents the highest possible temperature for the start of a martensitic transformation, where the Gibbs energy of austenite G_m_(B2-austenite) equals the Gibbs energy of martensite G_m_(martensite) with the same chemical composition. The observed *T*_0_ temperatures from the DSC experimental transformation features (see Table 2) are in reasonable accordance with the thermodynamic predictions, the latter suggesting 8 °C for *T*_0_(Ni_45_Ti_50_Cu_5_) and 77 °C for *T*_0_(Ni_40_Ti_50_Cu_10_), for the ortho-martensite.

The simulative trend of martensite phase stabilizations (see Figure 4b) from low to high Cu content basically agrees with the observed transformation sequence B19′↔B19′ + B19↔B19. It should be noted that the theoretic trends can only give a rough reproduction of experimental observations. In the case of HT-S1, even a partially amorphous state can be obtained by the chosen MA and SPS routine, for which “conservation” is coupled with very fast sintering rates [15,25,49]. Moreover, thermodynamics indeed postulate a chemically homogeneous material for each computation. With high heating rates in SPS, it is possible to traverse temperature regions very fast so as to minimize grain growth. However, when SMAs are produced using solid-state processing routes starting from powder mixtures, chemically homogeneous materials are difficult to obtain, due to the multitude of intrinsic (three types of atoms with different diffusion coefficients, high thermal conductivity of Cu and Ni or similar atomic radius of Ni, and Cu direct interactions between various species of atoms) and extrinsic (shape and size of powders, milling time, sintering temperature and sintering time) factors which act simultaneously [51]. As discussed, particularly in SMAs, even small heterogeneities likely have a strong effect on phase stabilities and thermal properties.

**Figure 12:**
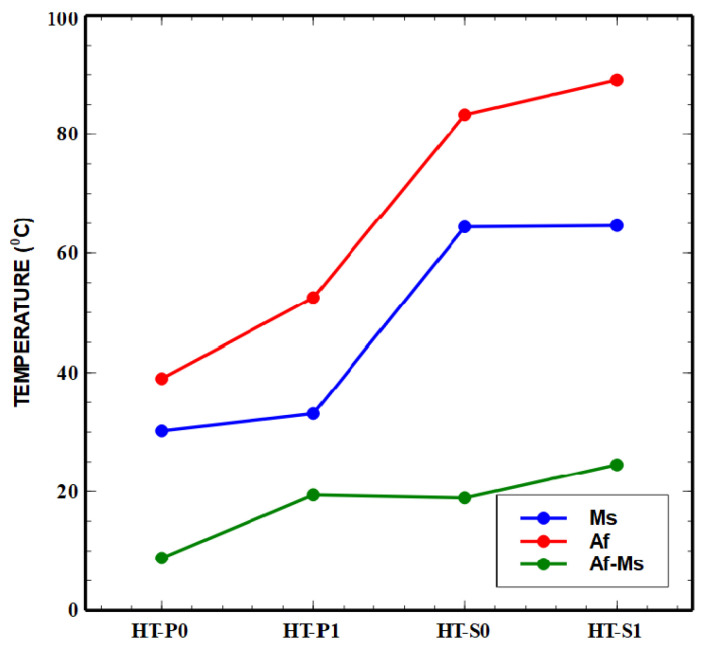
Behavior of Ms, Af and thermal hysteresis with the technological route with the mechanical alloying of NiTiCu.

The increase in the Cu content in the material led to the modification of the domain of the A↔M (or MT) phase transformation temperature from negative temperatures to positive temperatures. This is important, since positive transformation temperatures may allow for the easier realization of a shape memory behavior in biomedical applications. The role of the route of manufacturing for the hysteresis range and the temperatures of M_s_ and A_f_ of NiTiCu are indicated by the trends of plots presented in Figure 12, where M_s_ increases with an increase in Cu content in the alloy, which is comparable with the literature data [15,52].

Another important factor that had an influence on the MT was the mechanical alloying process applied in the manufacturing of NiTiCu materials. The reduced values of enthalpies in comparison to Goryczka et al. [9,50], who report an enthalpy between 12 and 17 J/g, may be a consequence of inhomogeneities of the chemical composition inside of the transformable phases and the presence of multiphase assemblies. The presence of multiphases was in fact identified by the XRD measurement, but this can only partially explain the enthalpy difference. 

The resulting values (Table 2) of the narrow present thermal hysteresis (A_f_-M_s_) are better than those identified in the literature [10,15], where for the alloys obtained by vacuum induction melting with 5% Cu alloying, the hysteresis was less than 20 °C, and for the alloys with 10% Cu alloying, the hysteresis yielded was 30 °C. A smaller hysteresis translates into a faster shape memory response to changes in temperature, which is especially important in thermo actuator applications of SMAs. In this context, it needs to be noted that for the mechanical alloying samples HT-P1 and HT-S1, thermal hysteresis increased from 9 °C to 19 °C for the alloys with 5% Cu and from 19 °C to 25 °C for the alloys with 10% Cu, which is in accordance with the literature [9,50].

#### 3.3.2. LFA Results

**Figure 13:**
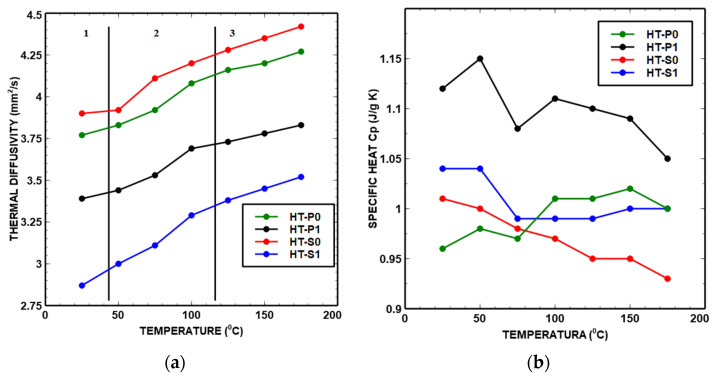
Thermal diffusivity (**a**) and specific heat (**b**) of NiTiCu shape memory alloys.

The trends of thermal key parameters *α*, *k* and *c*_p_ of the Ni_45_Ti_50_Cu_5_ and Ni_40_Ti_50_Cu_10_ as functions of temperature are shown in Figure 13. Table 3, Table 4, Table 5 and Table 6 show the data obtained from the laser flash analyzer.

**Figure 14:**
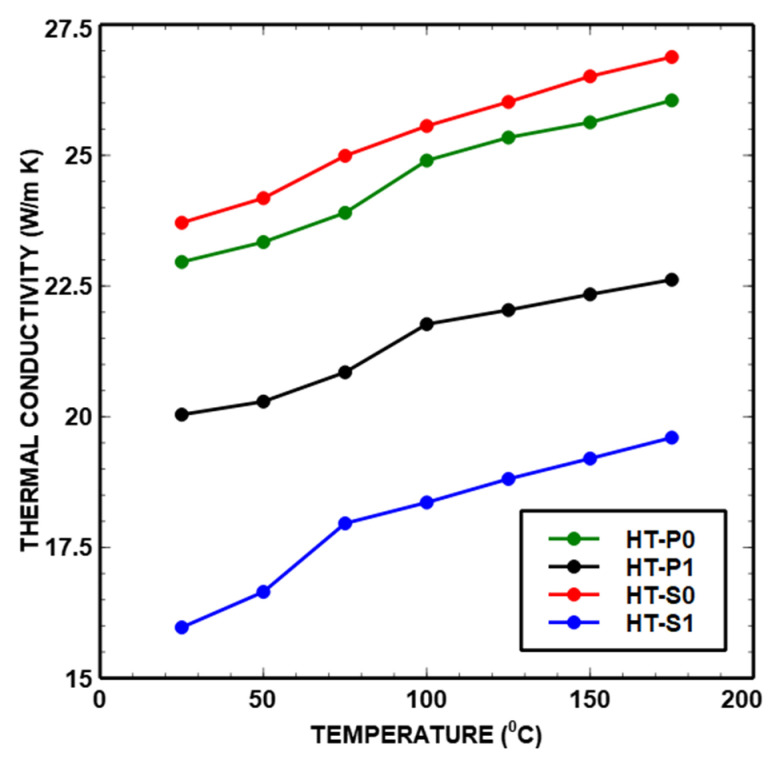
Thermal conductivity of NiTiCu shape memory alloys.

It can be seen from the trends of curves in Figure 13 and Figure 14 that there are several peaks due to the transitions in the region of 50 °C to 125 °C. The points where the patterns deviate from linear progression with an increase in temperature are referred to as inflection points. The points of inflection were observed at approximately 50 °C and 120 °C for all materials. As in *c*_p_ curves, the deviation is attributed to MT and precipitate phases and the associated structural reconfiguration towards a lower temperature that corresponds to the measured DSC curves (Figure 11). The structural reconfiguration is an effect of atoms’ redistribution which also causes a preferential orientation of the martensite phase [50].

- Thermal diffusivity

The temperature dependence of thermal diffusivity is revealed in Figure 13a. In our study, the thermal diffusivity tendency changed with temperature and Cu content. Three zones of different slopes of the thermal diffusivity curve can be identified, as indicated in Figure 13a. The slope of the thermal diffusivity curve had a positive value in zone 2 and was continuously linearly corresponding to the increase in temperature. Also, zone 2 combined the primary phases (austenite and martensite phases) and precipitated phases below 200 °C. The thermal diffusivities of the samples rapidly increased at a specific temperature range, due to heat transfer. The associated energy excited the metal atoms and increased the temperature of the metal.

An increase in the thermal diffusivity occurs at 80–100 °C and can correspond to the reverse MT of the M→A transformation, as identified by the DSC heating part, indeed with a small difference in the obtained austenite phase temperature between the two methods. One possible explanation for this difference could be the various rate of acquisition data measurement, because when the sample is heated to a specific temperature, the activation speed of the lattice atoms and associated lattice vibration and the movement of electrons will not be the same for different experimental setups. 

Both Cu-alloyed materials showed precipitation, with different fractions of each intermetallic Ni-Ti-based phase. Increasing the amount of Cu obviously led to less impedance against thermal conductivity and diffusivity, which is in agreement with the findings from the literature.

- Specific heat

The wavy *c*_p_–*T* curves from Figure 13b for all NiTiCu alloys indicate several transitions which occur with increasing temperature. It can be seen from Figure 13b that for HT-P0 and HT-P1, there are several peaks due to the transitions in the *c*_p_–*T* curve in the region of 80–175 °C. These peaks can be attributed to the structural rearrangement of atoms. In the case of HT-P0 and HT-P1, the area between 30 and 80 °C corresponds to reverse MT (i.e., M→A phase transformation) which increases the *c*_p_ up to 50 °C. Raising temperatures from 80 °C to 150 °C led to an alternation in the increase and decrease in *c*_p,_ which may be correlated with the appearance of a sequence of different precipitates. In the case of HT-S0 and HT-S1, the curves of *c*_p_ data reveal a peak at 50 °C that may indicate MT in the DSC heating part. All results of *c*_p_ are in accordance with the heating part of DSC curves presented in Figure 11.

- Thermal conductivity

It can be seen that the values of *k* (Figure 14) are in descending order from HT-S0 to HT-P0 to HT-P1 to HT-S1 (Table 3, Table 4, Table 5 and Table 6). This is mainly due to the increasing number of interfaces in a mechanically alloyed material. Moreover, when nanolamellar convoluted structures are inherited from MA during the subsequent alloy processing, this will have a strong effect on thermal conductivity [53,54]. This can further be correlated to the changing relative amounts of martensite phases, influenced by changing alloying and affected by complex precipitation. In HT-S0, the highest amount of the martensite stable phase was identified, which decreased with an increasing amount of precipitate phases of NiTi_2_ + Ni_2_Ti + Ni_3_Ti as shown by the experimental Ni-Ti-Cu phase stabilities (Figure 3). Even though the materials that present a cubic lattice, i.e., B2, NiTi_2_ and Ni_2_Ti, have been reported to exhibit a higher conductivity than more complex/lower symmetry crystalline forms including martensite, i.e., Ni_3_Ti, B19′ and B19 [34,35], the complexity of precipitation and interfaces seem to be more responsible for the observed trends of thermal properties than pure crystallographic aspects. In fact, the present behavior can be attributed to the complex combination of parameters with different weights—clearly important appears the level of porosity, which is quite smaller in HT-S0 and HT-S1, as compared to HT-P0 and particularly HT-P1. 

**Figure 15:**
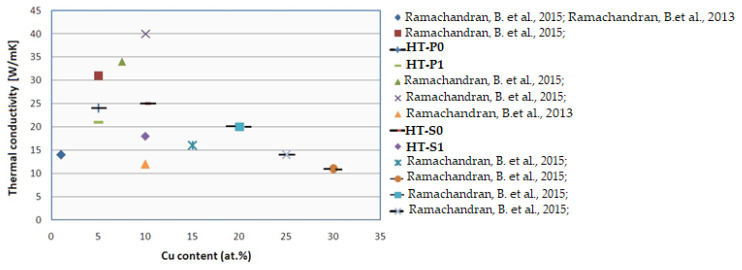
Variation in thermal conductivity with Cu content comparative with reference [20,21].

The determined *k* values (Table 3, Table 4, Table 5 and Table 6) are between 20.04 and 26.87 W/m K for HT-P0, HT-P1 and HT-S0 and are in agreement with the results identified for NiTiCu [20,21] presented in Figure 15, whereas only HT-S1 yielded a lower value of 15 W/m K for *k*, being closer to the values obtained for NiTi shape memory alloys processed by vacuum arc remelting [20]. 

To summarize, the factors that affect the thermal conductivity of alloys are thermo-mechanical treatment, alloy and phase compositions, the distribution and type of different phases and porosity. 

## 4. Discussion

### 4.1. Interrelation between Thermal Properties and Precipitation

The combination of the increased Cu content of 10% associated with the mechanical alloying (HT-S1) has a considerable impact on the thermal conductivity. One important aspect of thermal properties related to heterogeneous intermetallic precipitates is the overall excess interface area, which can be evaluated from the simulated precipitate sizes and number densities. We obtain around 470 mm^2^/mm^3^ and 3470 mm^2^/mm^3^ excess interface areas (smallest nano-particle population during the last cooling step, see Figure 10d, is neglected in this evaluation) due to the secondary precipitation of smaller particles in HT-P1 (overall around 5 mol.% Cu secondary precipitates) and HT-S1 (overall around 7 mol.% Cu secondary precipitates), respectively, with the simplified assumption of spherical precipitates. An order of magnitude larger excess interface area in Cu-rich HT-S1 than in HT-P1 is particularly produced by the considerable precipitation of two types of subgrain boundary particles, i.e., the nano-precipitates formed at the lamellae interfaces in amorphous or mixed amorphous/fine crystalline regions of MA samples. This will strongly contribute to the drop in thermal properties, which is obvious from Figure 12, Figure 13 and Figure 14. Further, the fact that two types of precipitates form at interfaces in HT-S1, as seen in Figure 10a–d, represents a higher complexity for interferences with thermal diffusivity and conductivity. This strongly suggests that not only the kinetic phase fraction but also the particle types, sizes and densities play an important role for microstructures—thermal properties’ relations. 

In MA-free HT-P0 and HT-S0 (Figure 9a–c), only a small fraction of small (radii around 15 nm) secondary precipitates, predominantly at dislocations around 1 mol.% (5% Cu) and only 0.2 mol.% (10% Cu), are predicted to evolve during aging. In fact, these particles also contribute to an excess interface region; however, they are distributed more homogeneously within the Ni-Ti matrix and thus may not interfere with thermal diffusivity in a comparable extent to heterogeneous precipitates at grain boundaries and nano-sized lamellae boundaries. Still, and consistent with thermal properties’ trends (see Figure 13 and Figure 14) from HT-P0 to HT-P1, these precipitates contribute to the slight increase in thermal diffusivity towards 10% Cu due to less obstacles. Towards higher Cu alloying, Ni_2_Ti is preferred over Ni_3_Ti precipitation in all cases in the simulation. However, one needs to take care by interpreting this trend in terms of the type of secondary Ni-Ti particles, since the respective thermodynamic stabilities of these phases lie close to each other, and even slight microstructural constraints may lead to the preferred precipitation of each of them. Moreover, simulated trends of changing particle fractions, number densities, sizes and precipitation sites (dislocations, grain boundaries and subgrain boundaries) as functions of alloying and thermo-mechanical treatment are robust. 

As mentioned, by mechanical alloying, a partially amorphous structure of HT-P1 and HT-S1 is induced. Whereas higher diffusivities in an amorphous phase [55] will directly increase thermal diffusivity, they will also promote precipitate evolution, with an opposite effect on thermal properties. Actually, the complex balance between these “counter-acting” mechanisms and the resulting influence on thermal conductivity cannot be evaluated precisely to date. Nevertheless, in this study, one side of the coin, which is the precipitate evolution, has been tackled. 

Fukuda et al. [56] investigated the transformation mechanism in Ti_50.5_Ni_44.5_Cu_5_ alloys by means of in situ TEM observation, electrical resistivity measurements and X-ray diffraction analysis, proposing a complex dependence of thermal conductivity on the type and changing amount of precipitation: a high fraction of NiTi_2_ precipitates led to the suppression of MT and also to the decrease in thermal conductivity, whereas an intermediate amount of precipitates led to an increase in thermal conductivity, especially for non-mechanically alloyed samples. On the other hand, NiTi_2_ precipitates assisted the decrease in thermal conductivity for mechanically alloyed samples. This suggests the different effects of (large) primary precipitates in MA-free processing, compared to (smaller) secondary precipitation in MA and SPS. An increase in thermal properties may indicate a change in the phonon scattering behavior with very large, isolated precipitates or a different role of the type of interface (grain boundaries and subgrain boundaries) for it. 

### 4.2. Interrelation between Thermal Properties and Density

**Figure 16:**
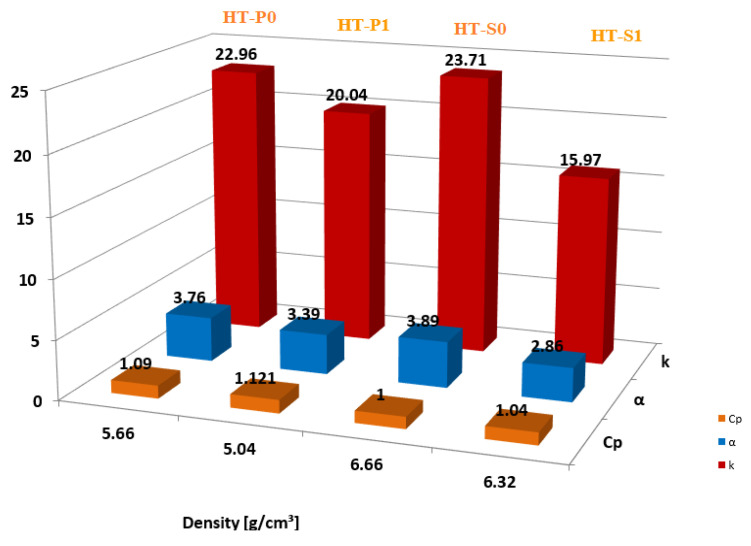
Evolution of thermal properties at 25 °C versus density of alloys.

Pore size, pore shape, pore orientation and the type of entrained gas are key factors that also lead to changes in thermal properties. In Figure 16, the thermal parameters at 25 °C versus the density of studied alloys are presented. From this figure, it is evident that decreasing *k* is related to porosity in the case of HT-P0 and HT-P1, and it increases with density vice versa, which is in accordance with the results of Zanotti et al. [57]. Due to decreasing porosity, when the temperature increases up to 150 °C, *k* increases by approximately 3 W/m K, because less air is entrapped within the materials. This can be explained by the heat transferred across the air space by an evaporation–condensation process [58].

**Figure 17:**
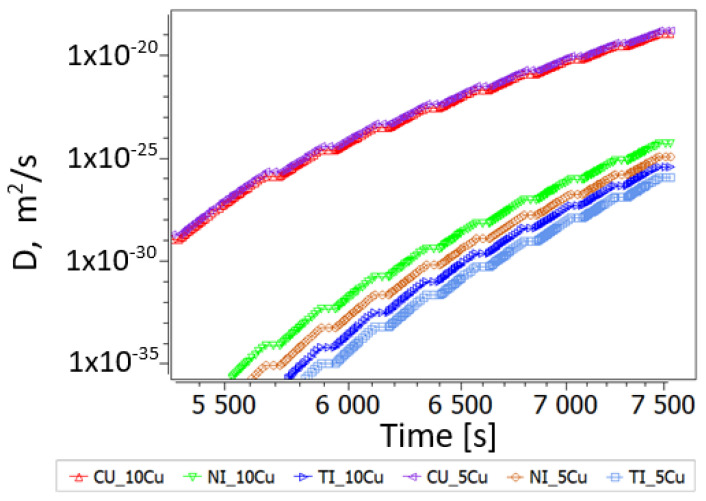
Diffusion of Ti, Ni and Cu in the B2 matrix during the heat treatment for LFA (after MA, SPS and aging). Diffusivities at 5Cu and 10Cu are almost identical. Steps in the curves reflect temperatures of resting for LFA shots.

In the case of Cu-rich samples HT-S0 and HT-S1, the porosities are quite similar, and the observed trend for *k* can thus not be explained by porosity alone. Whereas precipitate evolution at the relatively low measuring temperatures of LFA is negligible, the evaluated diffusion as function of temperature strongly increases for all alloying elements, Ti, Ni, and Cu (see Figure 17), which will be directly related to increased thermal diffusivity. Indeed, changes in diffusivities from 5 to 10 at.% Cu alloying are much smaller. Moreover, the predicted trend goes towards the faster diffusion of Ti and Ni in the B2 matrix with increased Cu alloying. 

Aside from direct precipitate effects on thermal properties, the diffusivity of Cu and Ni in Ti increases considerably for mechanically alloyed samples due to the composition evolution of the matrix.

The application of mechanical alloying led to a significant decrease in *k* and—particularly obvious in Cu-rich samples—α. These results are in accordance with the trends obtained for NiTi porous alloys manufactured by self-propagating high-temperature synthesis [19]. Taking into account the domain of MT temperature identified by DSC, we can attribute these values to the austenite phase, in accordance with the findings by D. Sharar et al. [58].

Possibly the temperature gradient in the heating/cooling processes during the manufacturing of the materials also contributes to the differences in thermal properties. Also, lattice distortion and low carrier concentration are also the main reasons for the increase in thermal conductivity with temperature in SMAs. The effect of Cu additions as a substitute for Ni in NiTi shape memory alloys (SMAs) on the delaying of its phase transition and narrowing hysteresis are well known, with NiTi-Cu SMAs having applications especially in the actuators that require such properties [8].

## 5. Conclusions

The present work shows that powder metallurgy and the use of the SPS technique can be successfully used to obtain NiTiCu alloys. The XRD difractograms show a partial crystalline structure, which was contained between 51.33 and 78.96, the highest crystallinity index being obtained for NiTiCu without MA.

The microstructure of NiTiCu is a mixture of primary phases like NiTi austenite (B2) abd NiTi martensite (B19′ and/or B19) and intermetallic phases, Ni_3_Ti/(Ni, Cu)_3_Ti, Ni_2_Ti/(Ni, Cu)_2_Ti and NiTi_2_/(Ni, Cu)Ti_2_. 

A thermodynamic Calphad assessment of the system is used in the thermokinetic prediction employing classical nucleation and growth modeling, in order to discuss the behavior of the multiphases identified by XRD analysis, which is considered to be crucial for the phase formations during the heating of MA material and SPS. The experimental findings can be correlated with thermodynamic metastability and thermokinetic simulation, when fluctuations in the Ti concentration of the 50:50 (at.%) balanced (Ni, Cu):Ti are postulated. A considerable effect of changing grain size and diffusion within the matrix due to MA and partial amorphization with the formation of shear bands on the precipitation during aging is predicted by the simulation and correlated with XRD, DSC and thermophysical properties.

The addition of Cu increased the M_s_ temperature from 30 to 60 °C and MT had the reversible sequence of austenite ↔ martensite. Transformation temperatures of MT and the thermal hysteresis of the reversible austenite ↔ martensite transformation depend on the composition and also on the manufacturing route of the alloys.

In this study, we identified the domain of MT temperatures between 34.6 and 73.85 °C for the NiTiCu without MA and 42.8 and 76.85 °C for the NiTiCu with MA. Increasing the Cu content up to 10 at.% leads to an increase in thermal hysteresis of about 20–25 °C. Thus, a small thermal hysteresis, confirm that the NiTiCu SMAs are promising for applications as sensors and/or actuators where a faster activation frequency is important.

Also, the thermal diffusivity, specific heat and thermal conductivity for Cu-alloyed NiTi-based SMAs were determined with varying Cu content and a process route with and without mechanical milling. The thermal conductivity and diffusivity of all alloys increased with temperature, whereas *c*_p_ curves showed a wavy, complicated trend as a function of temperature. The changing interfaces and associated varying fractions of precipitates at grain boundaries as functions of changing grain boundary lengths of refined grains due to MA correlate with trends of thermal properties. Moreover, variations in thermal properties were particularly seen for alloys with different porosities/densities, which may also be a consequence of changing phase content as a function of Cu alloying. The best thermal conductivity was obtained for NiTiCu without MA.

## Figures and Tables

**Table 1:** Setup for aging simulations. Other microstructural parameters are set as default (MatCalc presets).

	Dislocations, m/m^3^	Diffusion Factor	Grain Size, m	Sgb and Size (m)
Aging without MA after SPS	1 × 10^12^	1	50 × 10^−6^	none
Aging after MA and SPS	5 × 10^12^	10	10 × 10^−6^	0.1 × 10^−6^

**Table 2:** Transformation temperatures determined from DSC curves correlated with hardness from reference [6].

Sample	M_s_ [°C]	M_f_ [°C]	A_s_ [°C]	A_f_ [°C]	T_0_ = (M_s_ + A_f_)/2 [°C]	Thermal Hysteresis (A_f_ − M_s_) [°C]	HV_IT_ [6]
HT-P0	30.2	−40.1	−30.3	39	34.6	8.8	396
HT-P1	33.1	−2.7	−12.2	52.5	42.8	19.4	646
HT-S0	64.4	40.8	60.2	83.3	73.85	18.9	326
HT-S1	64.6	38.3	58.4	89.1	76.85	24.5	545

**Table 3:** Thermal conductivity, thermal diffusivity and specific heat of HT-P0.

Type	Temperature [°C]							
	25	50	75	100	125	150	175	
Density: 5.66 g/cm^3^ [6] Porosity: 9.19% [6]	22.96 ± 0.13	23.34 ± 0.15	23.90 ± 0.19	24.90 ± 0.04	25.34 ± 0.08	25.63 ± 0.13	26.05 ± 0.11	*k* [W/m K]
	3.77 ± 0.02	3.83 ± 0.02	3.92 ± 0.03	4.08 ± 0.01	4.16 ± 0.01	4.20 ± 0.02	4.27 ± 0.02	*α* [mm^2^/s]
	1.09 ± 0.01	1.08 ± 0.01	1.18 ± 0.24	1.05 ± 0.01	1.03 ± 0.01	1.01 ± 0.01	1.21 ± 0.01	*c*_p_ [J/g K]

**Table 4:** Thermal conductivity, thermal diffusivity and specific heat of HT-P1.

Type	Temperature [°C]							
	25	50	75	100	125	150	175	
Density: 5.04 g/cm^3^ [6] Porosity: 18.73% [6]	20.04 ± 0.05	20.29 ± 0.11	20.85 ± 0.09	21.77 ± 0.07	22.04 ± 0.02	22.34 ± 0.07	22.62 ± 0.07	*k* [W/m K]
	3.39 ± 0.01	3.44 ± 0.01	3.53 ± 0.01	3.69 ± 0.01	3.73 ± 0.01	3.78 ± 0.01	3.83 ± 0.01	*α* [mm^2^/s]
	1.12 ± 0.01	1.15 ± 0.07	1.08 ± 0.07	1.11 ± 0.01	1.10 ± 0.01	1.09 ± 0.01	1.05 ± 0.02	*c*_p_ [J/g K]

**Table 5:** Thermal conductivity, thermal diffusivity and specific heat of HT-S0.

Type	Temperature [°C]							
	25	50	75	100	125	150	175	
Density: 6.66 g/cm^3^ [6] Porosity: 3.29% [6]	23.71 ± 0.33	24.18 ± 0.12	24.99 ± 0.01	25.56 ± 0.15	26.02 ± 0.14	26.51 ± 0.10	26.88 ± 0.09	*k* [W/m K]
	3.90 ± 0.05	3.92 ± 0.01	4.11 ± 0.01	4.20 ± 0.02	4.28 ± 0.02	4.35 ± 0.01	4.42 ± 0.01	*α* [mm^2^/s]
	1.01 ± 0.02	1.00 ± 0.01	0.98 ± 0.03	0.97 ± 0.01	0.95 ± 0.02	0.95 ± 0.01	0.93 ± 0.01	*c*_p_ [J/g K]

**Table 6:** Thermal conductivity, thermal diffusivity and specific heat of HT-S1.

Type	Temperature [°C]							
	25	50	75	100	125	150	175	
Density: 6.32 g/cm^3^ [6] Porosity: 2.72% [6]	15.97 ± 0.02	16.65 ± 0.10	17.96 ± 0.01	18.36 ± 0.07	18.81 ± 0.06	19.20 ± 0.07	19.60 ± 0.06	*k* [W/m K]
	2.87 ± 0.01	3.00 ± 0.01	3.11 ± 0.01	3.29 ± 0.01	3.38 ± 0.01	3.45 ± 0.01	3.52 ± 0.01	*α* [mm^2^/s]
	1.04 ± 0.02	1.04 ± 0.01	0.99 ± 0.01	0.99 ± 0.01	0.99 ± 0.02	1.00 ± 0.01	1.00 ± 0.02	*c*_p_ [J/g K]

## Data Availability

Data are contained within the article.
